# An automated solution for measuring the progress toward FAIR research data

**DOI:** 10.1016/j.patter.2021.100370

**Published:** 2021-10-29

**Authors:** Anusuriya Devaraju, Robert Huber

**Affiliations:** 1 Terrestrial Ecosystem Research Network (TERN), The University of Queensland, Long Pocket Precinct, Level 5 Foxtail Building #1019, 80 Meiers Road, Indooroopilly, QLD 4068 Australia; 2 Center for Marine Environmental Sciences (MARUM), University of Bremen, Leobener Strasse 8, 28359 Bremen, Germany

**Keywords:** FAIR data principles, research objects, metrics, automated assessment, trustworthy digital repository, data reuse, data discovery

## Abstract

With a rising number of scientific datasets published and the need to test their Findable, Accessible, Interoperable, and Reusable (FAIR) compliance repeatedly, data stakeholders have recognized the importance of an automated FAIR assessment. This paper presents a programmatic solution for assessing the FAIRness of research data. We describe the translation of the FAIR data principles into measurable metrics and the application of the metrics in evaluating FAIR compliance of research data through an open-source tool we developed. For each metric, we conceptualized and implemented practical tests drawn upon prevailing data curation and sharing practices, and the paper discusses their rationales. We demonstrate the work by evaluating multidisciplinary datasets from trustworthy repositories, followed by recommendations and improvements. We believe our experience in developing and applying the metrics in practice and the lessons we learned from it will provide helpful information to others developing similar approaches to assess different types of digital objects and services.

## Highlights

• A set of core metrics for assessing the FAIR compliance of scientific data
• An automated assessment tool for applying the metrics in practice
• FAIR assessments are drawn upon widely adopted data sharing practices
• Tested and used in the context of diverse data repositories

## The bigger picture

In recent years, there has been a strong recommendation from funders, publishers, and research organizations on adopting the Findable, Accessible, Interoperable, and Reusable (FAIR) principles to maximize scientific data availability and reuse. However, measuring the FAIRness of research data in practice is challenging. The number of datasets has proliferated in scientific research, and new datasets are emerging from various sources. This paper aims to contribute to the FAIR data assessment through a set of core metrics elaborating the principles and an automated tool that supports FAIR evaluation based on the metrics. The metrics are built on established work and consider standard data practices. The tool development is collaborative and iterative. The consultative process has motivated the repositories to refine their data services. Further pilots are planned with the repositories in the European Open Science Cloud. The broader goal is to adapt the solution to assess other digital objects such as vocabularies and software.

## Teaser

One of the open questions around the FAIR assessment is how to measure FAIRness of the scientific data in practice. We found that explicit and machine-assessable metrics drawn upon prevailing data sharing practices are vital to evaluating FAIR data programmatically. An automated FAIR assessment is continuous development and must go beyond the object itself. FAIR-enabling repositories should evolve in parallel to support continued access to and long-term preservation of FAIR data.

## Introduction

### Motivation

Providing long-term preservation and continued access to research data^1^ is vital to support researchers and potential users to fully leverage the data to address social, economic, and environmental problems. The goal of the Findable, Accessible, Interoperable, and Reusable (FAIR) principles is to guide data producers, providers, and practitioners to share research data in a way that will maximize their reuse.^2^ Funders, publishers, data service providers, and policy makers have strongly endorsed adopting the principles to digital objects such as research data,^3^ since the principles were published in 2016.^2^ However, assessing the FAIR compliance of both repositories and data objects is difficult since appropriate practical solutions are either missing or not fully developed.^3^ Further, there are several challenges when translating the FAIR principles to quantifiable criteria and applications. Some of the FAIR principles are rather vaguely described.^3^, ^4^, ^5^, ^6^ Further, many of these principles are based on tacit knowledge (“rich metadata,” “persistent identifiers”) instead of clear, verifiable indicators. Consequently, to date, much of existing work on FAIR assessment focuses on what needs to be measured, which led to the development of indicators (also called metrics or criteria) elaborating the principles; e.g., Research Data Alliance (RDA) FAIR Data Maturity Model.^7^

One of the open questions around the FAIR assessment has been how to measure the FAIRness of the scientific data in practice.^4^ Several manual assessment tools (as provided in the section “related work”) have been piloted but are primarily intended to engage and educate research communities to make their data FAIR. Notably, the manual assessment approach is not scalable, considering a vast volume of data curated at data repositories, which may proliferate with future deposits. This indicates a strong need for automated data evaluations.^4^^,^^8^

**Figure 1:**
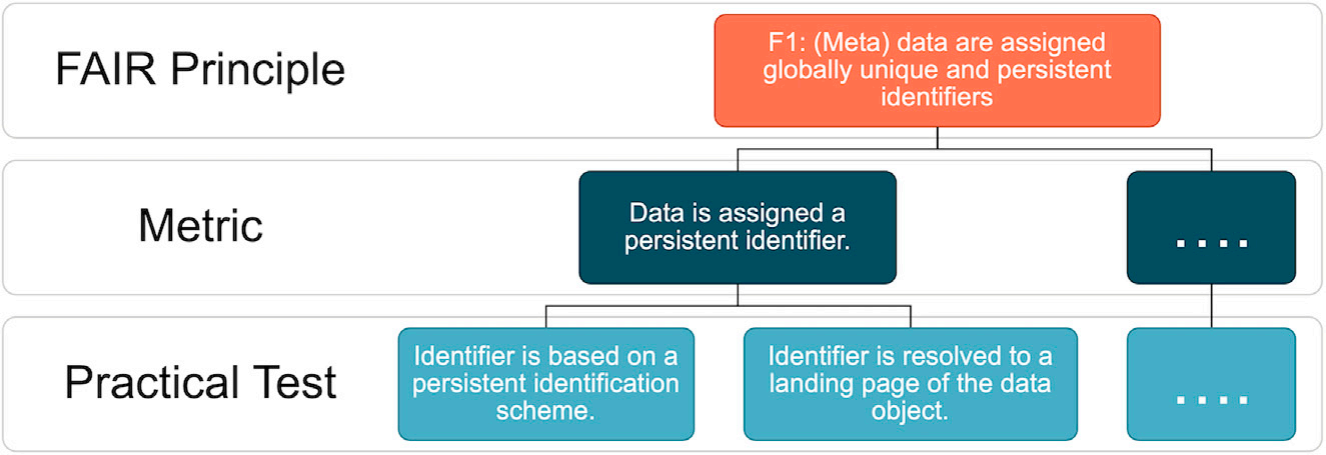
An example of a principle, its metric, and practical tests

This paper offers two essential contributions to the FAIR assessment of research data objects: a set of core quantifiable FAIR metrics and an open-source tool, F-UJI, that applies the metrics to measure the progress of FAIR aspects of data programmatically.^9^ Throughout this paper, the term “data object” refers to research data. “Core metrics” refers to the domain-agnostic assessment criteria that are centered on generally applicable metadata and data characteristics. The paper describes the practical tests implemented in the tool against the metrics and the rationales behind the tests. Figure 1 illustrates the relation between the FAIR principles, metrics, and tests. We recognize that automated assessment depends on explicit, machine-accessible criteria, and FAIR evaluation subject to data practices of various communities. This paper elaborates how we design and implement the tests following existing best practices and standards in research data preservation and publication, and as reflected in the literature. We believe this work substantially complements current work on FAIR metrics (e.g., RDA FAIR Data Maturity Model), which lacks details of practical tests to apply the metrics in practice.

The tool and its underlying metrics are significant outputs of the Horizon 2020 FAIRsFAIR project,^10^ which focuses on building practical solutions to support the application of FAIR principles throughout the research data life cycle in the European Open Science Cloud (EOSC). In fact, the EOSC FAIR Working Group informs the metrics as one of two existing developments relevant to the definition of FAIR metrics for data in the context of EOSC.^8^ We have tested F-UJI extensively with the pilot data repositories selected for in-depth collaboration with the FAIRsFAIR project. Beyond the project, the tool is currently actively tested and applied by various EOSC projects and data service providers. Unlike existing approaches that focus solely on the FAIR assessment, our work incorporates an iterative consultation with data repositories as part of the tool development. The consultation comprises rounds of user feedback to refine the tool and metrics and has motivated the pilot repositories to improve their data services.

We organize the paper as follows: the section “related work” presents related work, followed by an overview of iterative development (section “development approach"). Section “fair data object assessment metrics” describes the FAIR metrics, including their practical tests and rationales. Section “technical implementation” covers the tool's implementation; testing and results are specified in section “evaluation and results.” Section “discussions” discusses the importance, relevance, and the lessons learned from the work, and finally the section “conclusions” highlights its critical areas of improvement.

### Related work

Several tools have been developed to assess FAIR data; see FAIRassist.org^11^ and the RDA FAIR Data Maturity Working Group survey on existing FAIR assessment tools.^12^ Most of the existing work focuses on the manual FAIR data assessment, carried out through case studies,^13^ checklists, and templates; e.g., ARDC self-assessment tool,^14^ FAIR-Aware,^15^ SHARC IG FAIR Template,^16^ and WDS/RDA Assessment of Data Fitness for Use checklist.^17^

To date, there have been limited computational approaches to evaluating data FAIRness. Notable work in this direction is the FAIR Evaluation Services,^5^ which assesses data programmatically based on maturity indicators. FAIRshake is a toolkit that facilitates the manual assessment of biomedical digital objects (e.g., datasets, tools, and databases) based on existing or user-defined metrics (also called rubrics).^18^ The toolkit utilizes the structured data available on the selected object pages to perform automated assessments. An essential feature of the toolkit is that it allows users to submit a manual FAIR assessment of a biomedical digital object from the object's homepage to the FAIRshake site and visualize the evaluation through a browser extension. A semi-automated workflow was developed by Ammar et al.^19^ to demonstrate FAIR data assessment in life sciences repositories. Some focus on measuring specific aspects of FAIR; e.g., DataONE service quantitatively evaluates the metadata completeness and effectiveness.^20^ The work we specified in this paper has a similar goal as the developments above, which is enabling a programmatic FAIR data assessment, but it has specific focus. Current work aims at facilitating FAIR evaluations of various objects (software, data, repository), and, in some cases, to serve a specific research community (e.g., FAIRshake). In contrast, the scope of our work has been refined to meet the demands of various service providers (e.g., data repositories, data portals, or registries) committed to FAIR data provision. These providers require a practical solution to programmatically measure their datasets for their level of FAIRness over time. There are several important aspects in which we believe our approach makes important contributions to FAIR assessment:

• The work aligns with the recommendation developed by the EOSC FAIR Working Group (see recommendation 3 on the Definition and Implementation of Metrics).^8^ It centered on the core metrics drawn upon the RDA FAIR Data Maturity Model Working Group criteria.
• To support in-depth data assessment, F-UJI interfaces with FAIR-enabling services; e.g., re3data,^21^ SPDX license registry,^22^ and RDA metadata catalog.^23^
• To promote the broader application of our work, the practical tests against the metrics are implemented following common data standards and practices.
• The tool's development process incorporates a consultative process with data repositories (more details in section “evaluation and results”). Consultation includes interpreting the assessment results to identify critical areas of FAIR data improvement and action plan moving forward. The approach helps in developing a tool that meets actual data repositories needs.

### Development approach

**Figure 2:**
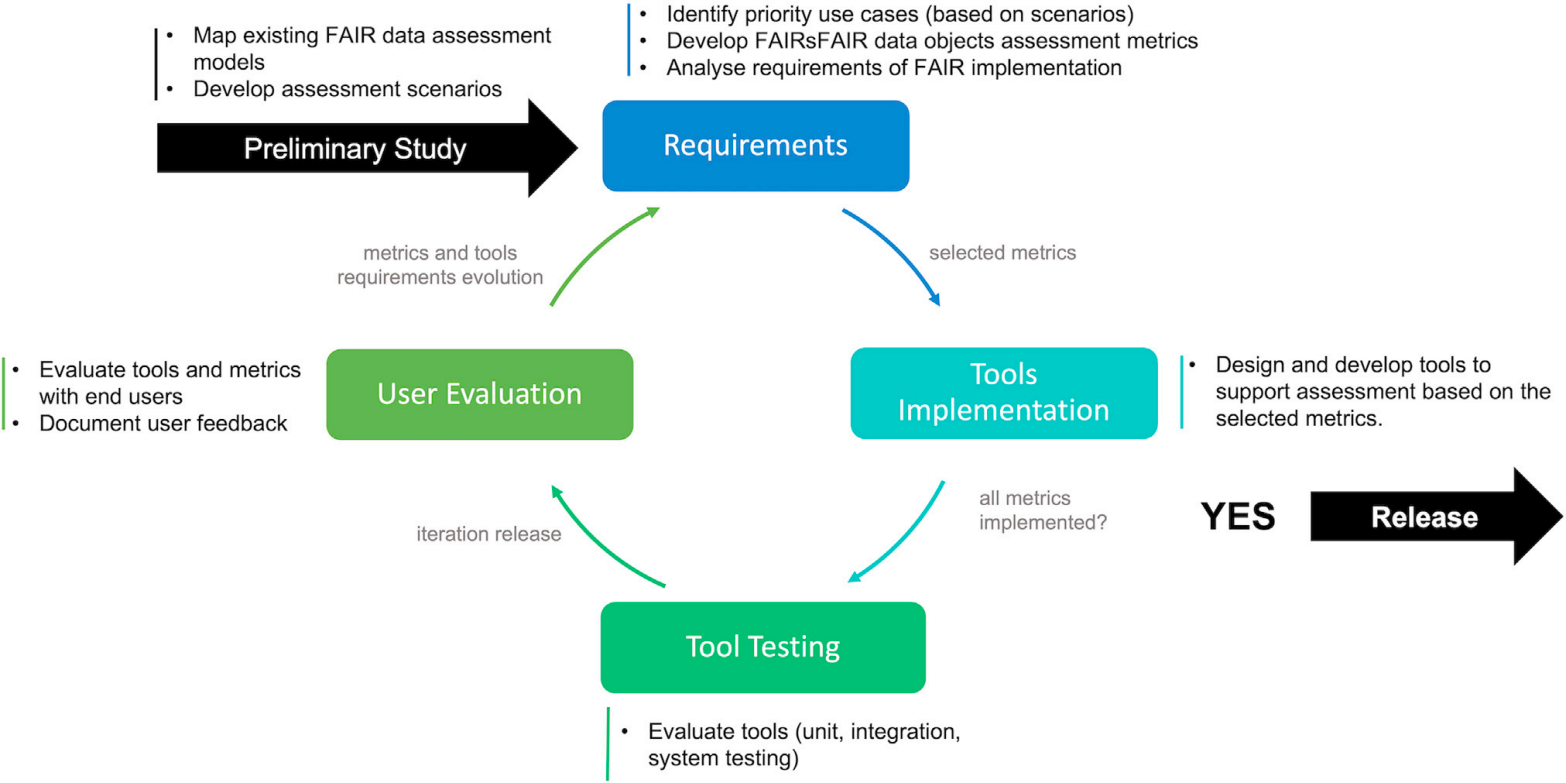
Iterative development of F-UJI

Figure 2 illustrates the iterative development. During the preliminary study, we explored several FAIR assessment scenarios^24^ and reviewed existing FAIR assessment frameworks. One of the outcomes of the study was draft metrics^6^ of FAIR data assessment. During requirements analysis, we finalized the use case of interest and identified its early adopters (i.e., pilot repositories), and developed the specification of the metrics, including practical tests. The use case focuses on enabling trustworthy data repositories committed to FAIR data provision to programmatically measure datasets for their level of FAIRness over time. We conceptualized and implemented the tests representing the metrics, performed system testing, and then evaluated the implementation with the repositories. The feedback from the repositories was used as a basis to improve the tool (and the metrics) in succeeding iterations.

### FAIR data object assessment metrics

We formulated 17 core metrics to support a systematic assessment of FAIR data objects (Table 1). The FAIR principles are proposed as “domain-independent, high-level principles that can be applied to a wide range of scholarly outputs.”^2^ As we aimed for the broadest possible interdisciplinary application scenario, we focus on FAIR assessment following the domain-agnostic Web standards, e.g., Dublin Core,^25^ DataCite Metadata Schema,^26^ Schema.org,^27^ and OpenGraph,^28^ and standards commonly used within the scientific community, such as Data Catalog Vocabulary (DCAT-2),^29^ Metadata Encoding and Transmission Standard,^30^ Metadata Object Description Schema (MODS),^31^ Ecological Metadata Language,^32^ ISO19139 Geographic information - Metadata,^33^ and Data Documentation Initiative (DDI) Codebook.^34^

**Table 1:** FAIRsFAIR object assessment metrics and practical tests

Principle	Metrics	Practical tests
F1	FsF-F1-01D data are assigned a globally unique identifier	• The object should be given a unique identifier (URI) that follows a proper syntax • The identifier is Web accessible (not broken)
F1	FsF-F1-02D data are assigned a persistent identifier	• A data identifier is specified based on a commonly accepted persistent identifier scheme suitable for digital objects • The identifier resolves to a landing page with metadata of the data object
F2	FsF-F2-01M metadata include descriptive core elements to support data findability	• Limited core metadata properties are specified • Citation metadata properties are specified (creator, title, publication date, publisher, identifier, resource type) through appropriate metadata fields • All minimum descriptive metadata properties (creator, title, publisher, publication date, summary, keywords, identifier, resource type) are specified through appropriate metadata fields
F3	FsF-F3-01M metadata include the identifier of the data they describe	• Metadata contain a PID or URL that represents the Web location of the downloadable data content • A data identifier is included in the metadata, and it matches the identifier provided as part of the assessment request
F4	FsF-F4-01M metadata are offered in such a way that they can be retrieved by machines	• Metadata of the object are retrievable programmatically through at least one of the following methods: ○ structured data embedded in the landing page of the data object ○ typed links of metadata document or signposting header links ○ content negotiation with a PID provider service
A1	FsF-A1-01M metadata contain access level and access conditions of the data	• Metadata include the level of data access (e.g., public, embargoed, restricted) and their access conditions using appropriate metadata fields • Access level metadata are machine readable, and this is verified against controlled vocabularies (COAR, Eprints, EU Vocabulary, and OpenAIRE)
A1	FsF-A1-02M metadata are accessible through a standardized communication protocol	• The metadata URI's scheme is based on a common application protocol • The metadata are accessible through the identifier provided
A1	FsF-A1-03D data are accessible through a standardized communication protocol	• The data URI's scheme is based on a shared application protocol • The data are accessible through the identifier provided
A2	FsF-A2-01M metadata remain available, even if the data are no longer available	• Preservation of data and metadata is an explicit role of the repository. Therefore, it should be assessed at the level of a repository, not at the level of individual objects. For more information, see section “metadata preservation (FsF-A2-01M)”
I1	FsF-I1-01M metadata are represented using a formal knowledge representation language	• The metadata of the object are available in a formal knowledge representation language; e.g., through at least one of the following mechanisms: ○ parsable, structured data are embedded in the landing page ○ parsable, formal metadata (e.g., RDF, JSON-LD) are accessible through content negotiation, typed links, or SPARQL endpoint
I1	FsF-I1-02M metadata use semantic resources	• Namespaces of known semantic resources (excluding common namespaces; e.g., RDF, RDFS, XSD, OWL) are present in the metadata of an object
I3	FsF-I3-01M metadata include links between the data and its related entities	• Metadata capture the relation between a data object and its related entity. The relation should be expressed using a relation type and, if a URI is used to represent a related entity, it should be accessible
R1	FsF-R1-01MD metadata specify the content of the data	• Metadata include the type of the object and the technical properties of its data file, such as format, size, and observed variables • Metadata values of the properties comply with the actual data file
R1.1	FsF-R1.1-01M metadata include license information under which data can be reused	• Metadata contain license information represented using an appropriate metadata element • A standard, machine-readable license is specified
R1.2	FsF-R1.2-01M metadata include provenance information about data creation or generation	• Metadata include properties representing data creation, such as creator, contributors, creation and modification dates and version, source, and relations that indicate data creation activities • Provenance metadata are available in a machine-readable version of PROV-O or PAV
R1.3	FsF-R1.3-01M metadata follow a standard recommended by the target research community of the data	• Metadata are available through at least one of the domain metadata standards listed in the RDA Metadata Standards Catalog
R1.3	FsF-R1.3-02D data are available in a file format recommended by the target research community	• Data are available in a long-term file format as defined in ISO/TR 22299^35^ • Data are available in an open format • Data are available in a scientific file format (e.g., Library of Congress dataset formats, Wolfram Alpha supported file formats)

We built the metrics based on the indicators proposed by the RDA FAIR Data Maturity Model Working Group^7^ and on prior work conducted by the project partners. We have improved the metrics based on the feedback from FAIR stakeholders through various means; e.g., a focus group study, open consultation, and pilot repositories. For more details on the methodology, see Devaraju et al.^24^ The metrics (v0.4) specified by this paper are detailed in the specification.^36^

#### Hierarchical model

FAIR principles are high-level guidelines. To evaluate datasets based on the principles objectively, we clarified each of the principles in terms of one or more metrics. A metric can be tested in various means, depending on contexts (e.g., repository and domain requirements, data types, and formats). Hence, we developed one or more practical tests detailing the metric. For example, in Figure 1, one of the metrics representing the principle F1 focuses on the persistence identification of data. This metric will be evaluated in terms of two practical tests. The first test verifies if the data identifier is based on a persistent identifier (PID) scheme. The second test checks if the identifier resolves to a digital resource (i.e., a landing page) of the object. The hierarchical model (principle-metric-practical test) offers several benefits. Individual new tests can be developed against the metrics and tailored to community requirements. Assessment scores resulting from the tests can be aggregated to communicate the FAIRness of a dataset, either by principle or metric, as appropriate.

Each metric is uniquely identified following the extended Backus–Naur form (EBNF) notation below:

FsFMetricLabel:: = “FsF-”([FAIR][0–9](“-”[0–9])?) (“- ”[0–9]+) (“M”| “D”|“MD”)

For example, the unique identifier of the metric FsF-F1-01D starts with the shortened form of the project's name (FAIRsFAIR), followed by the FAIR principle (F1) and the local code of the metric. The last part of the identifier refers to the resource that will be evaluated based on the metric; e.g., data (D), metadata (M), or both (MD).

#### Practical tests and rationales

In this section, we provide essential insights into developing the metrics and their practical tests. We consolidate closely related metrics into a particular sub-section. For example, section “object identification (FsF-F1-01D, FsF-F1-02D)” covers both metrics (on persistence and uniqueness) that represent object identification. For a summary of all metrics and tests, see Table 1. Technical details are provided in section “technical implementation.”

##### Object identification (FsF-F1-01D, FsF-F1-02D)

When evaluating an object's identifier, we make a distinction between its uniqueness (FsF-F1-01D) and persistence (FsF-F1-02D). For example, a Uniform Resource Locator (URL) or Universally Unique Identifier (UUID) is globally unique but not necessarily persistent. Widely used persistent identifiers, e.g., DOI, Handle, PURL, w3id, ARK, are maintained and governed such that they remain stable and resolvable for a long term. The tests evaluate if the object's identifier follows a proper syntax (i.e., based on a persistence identifier scheme) and if it successfully resolves to a landing page that contains metadata of the object. Here, our metrics differ from criteria in, e.g., the RDA FAIR data maturity model since we follow the current best practice to have a PID resolving to a single landing page^37^ instead of requiring that a PID be registered separately to both data and metadata of an object.

##### Descriptive core metadata (FsF-F2-01M)

The rich metadata required to support data discovery depend on the discipline and applications. Our work focuses on descriptive metadata,^38^^,^^39^ particularly core metadata properties enabling data citation and discovery. Following the existing citation guidelines,^40^, ^41^, ^42^, ^43^ we identified relevant metadata properties for citing data, such as creator, title, publication date, publisher, and identifier. In addition, the summary or description is vital as it usually summarizes data contexts, and keywords help search applications. Therefore, we regard these as minimum descriptive metadata to support data findability. Further, we followed Fenner et al.’s^43^ recommendation for obligatory inclusion of the resource type within the metadata since this allows us to distinguish research data from other digital objects. These metadata properties align well with recommendations for data discovery and core metadata definition.^26^^,^^44^^,^^45^ They represent a good intersection between sets of metadata elements used by domain-agnostic metadata standards Dublin Core, DCAT-2, Schema.org, and DataCite metadata schema.

##### Inclusion of data identifiers (FsF-F3-01M) and data content descriptors (FsF-R1-01MD)

One of the interests of the research community is the downloadable link of a data object's contents. Most domain-agnostic standards (e.g., DataCite metadata schema, Schema.org [https://schema.org/Dataset], and DCAT) include specific properties for representing data content identifiers that we use to verify the metric FsF-F3-01M. In addition, we evaluate if an object's metadata include its data and content identifiers through other recognized mechanisms; e.g., the typed links adapted by Sompel and Nelson^46^ in their FAIR signposting profile.

The practical tests against the metric FsF-R1-01MD evaluate if the object's metadata include the content description of the object, and the description reflects the actual data files, which we retrieved through the content identifiers supplied in the metadata. We determine the content descriptors based on RDA's WDS/RDA Assessment of Data Fitness for Use Working Group's recommendations. It covers the technical properties such as file format and size, and variable(s), which can help data processing and reuse. The future expansion of the content descriptors depends on some agreed mechanism for defining domain requirements and evolving data interoperability solutions such as the data-package standard supported by Frictionless Data^47^ and W3C CSV on the Web recommendations.^48^

##### Searchable metadata (FsF-F4-01M)

Assessing a data object based on the metric FsF-F4-01M will require an understanding of the data access services supported by its repository. A data provider may disseminate data in several ways, for example, through a proprietary Web service, and harvesting protocols (e.g., Open Archives Initiative Protocol for Metadata Harvesting [OAI-PMH]^49^ and Catalog Service for the Web [CSW]),^50^ which may not necessarily use the same identifier as the one used to publish the data object.^51^ Today's searchability of data objects largely depends on the use of Web standards supported by major search engines. Data providers embed structured data in the data landing pages^51^^,^^52^ for use by search engines. Within the research data community, content negotiation is a W3C recommended practice for providing data on the Web^44^ and currently gains considerable momentum within the open data community through the emerging ”content negotiation by profile” approach.^53^ Another practice is providing typed links representing scholarly metadata.^46^ Therefore, we implemented the tests based upon the three common practices (structured data, content negotiation, and typed links) to verify if an object's metadata is discoverable by machines; for technical details, see section “identifier-based metadata extraction.”

##### Data accessibility (FsF-A1-01M, FsF-A1-02M, FsF-A1-03D)

The accessibility principle (A1) of FAIR emphasizes standardized communication protocol, authentication, and authorization for accessing data. In addition to these, it is vital to capture metadata related to data access so that machines and humans comprehend the access requirements and then can use them to retrieve the data accordingly. For this reason, the practical test of the metric FsF-A1-01M determines if the metadata includes the data access level (e.g., public, embargoed, restricted) and its access conditions using appropriate metadata fields. To ensure access levels are machine understandable, the test also verifies if the access level information is expressed using controlled vocabularies such as the Confederation of Open Access Repositories (COAR),^54^ Eprints,^55^ EU Vocabulary,^56^ and OpenAIRE access rights.^57^

We further evaluate the object if its metadata (FsF-A1-02M) and data (FsF-A1-03D) are accessible through a standard communication protocol. Although there are many application-layer protocols, metadata and data should not be disseminated using proprietary or outdated protocols (e.g., Apple Filing Protocol, Gopher) to encourage data reuse. Tests against these metrics use regular expressions to extract the Uniform Resource Identifier (URI) scheme of the object's URI and verify if the scheme corresponds to shared application-layer protocols. The resulting relatively short, extensible, hand-curated list of URI schemes^58^ includes (s)HTTP(s), (s)FTP, SSN, SVN, telnet, RTSP, and WS(s).

##### Metadata preservation (FsF-A2-01M)

Programmatic assessment of the preservation of metadata of a data object can only be tested if the object is deleted or replaced. Therefore, this test is only applicable for deleted, replaced, or obsolete objects. Importantly, continued access to metadata depends on a data repository's preservation practice. Therefore, we regard that the assessment of metric applies at the level of a repository, not at the level of individual objects. For this reason, we excluded this metric from the implementation.

##### Semantics interoperability (FsF-I1-01M, FsF-I1-02M)

The I1 principle of FAIR loosely defines the use of knowledge representation. Hence, we represented two metrics expressing the principle. The metric FsF-I1-01M focuses on making the metadata available in a knowledge representation language (e.g., RDF, RDFS, OWL). The metric FsF-I1-02M is the continuation of the assessment FsF-I1-01M. However, it focuses on using semantic resources (e.g., ontology, thesaurus, and taxonomy) to describe the metadata contents unambiguously. For example, the metadata of isotope data from the International Ocean Discovery Program (IODP) may be available in the RDF graph (e.g., serialized in RDF/XML). In contrast, the graph uses the World Register of Marine Species (WoRMS) to express the species observed. We assume that the namespaces of semantic resources in a metadata document indicate that metadata uses the resources. Therefore, the test compares the namespaces extracted from the metadata document with entries in services for semantic resources. We exclude generic namespaces (e.g., of RDF, RDFS, and XSD) from the comparison. There is no well-maintained, cross-domain semantic resources registry available yet; therefore, we consolidate two sources, Linked Open Vocabularies (LOV) and Linked Open Data Cloud, to support namespace comparison; deprecated semantic resources were excluded as part of this process. Until an authoritative, well-maintained list of namespaces of semantic resources is developed, we regard this as an interim solution to verify the presence or absence of used semantic resources.

The I2 principle of FAIR focuses on FAIR vocabularies. At present, it is hard to verify if the metadata uses FAIR vocabularies as the criteria defining a FAIR vocabulary have not been fully developed and recommended yet. For this reason, Table 1 does not include relevant metrics on this principle. Ongoing developments on enabling FAIR vocabularies are by Cox et al.,^59^ Jonquet et al.,^60^ and Le Franc et al.^61^

##### Linked related resources (FsF-I3-01M)

A data object may be linked to related entities; e.g., its prior version, publications, funder, and platform. The practical test of FsF-I3-01M requires a link between a data object and its related entity to be expressed using a relation type and related entity, for ease of interpretation and comparison. In practice, a data object may be linked to an N number of entities. Therefore, the practical test does not weigh the evaluation criteria based on the quantity or types of relations. While this is not an absolute requirement of FsF-I3-01M, as part of future work, persistent identifiers should be preferable when representing related entities (e.g., Open Researcher and Contributor ID [ORCID] for contributors, Digital Object Identifier [DOI] for publications, International Geo Sample Number [IGSN] for physical specimens, and Research Organization Registry [ROR] for institutions) to link identifiers in a standardized way through a PID graph or expressed them through signposting links.^46^

##### Data usage license (FsF-R1.1-01M)

We encourage the use of licenses for all kinds of data, whether public or restricted. Without an explicit license, users do not have a clear idea of the data's legal context. The test associated with the metric FsF-R1.1-01M checks if a data object's license is specified using an appropriate metadata field. It uses the SPDX License List to verify the name and the type of the license specified.

##### Provenance of data creation (FsF-R1.2-01M)

Metadata on data provenance can vary depending on the data stage (i.e., from creation to publication and then usage), type, purpose, and application.^62^^,^^63^ For this reason, it is laborious to define a set of finite provenance properties that will be adequate for all use cases and disciplines. Therefore, we focus on the provenance of data creation as specified by Miles et al.^64^ For instance, the test evaluates what, who, when, and how aspects of data generation or collection. In detail, examples of the provenance properties include data sources (e.g., their previous release and instruments), data creation or collection date, contributors involved in data creation and their roles and data publication, modification, and versioning information. Since the FAIR principles emphasize machine actionability,^3^ we evaluate if the provenance metadata are accessible in a machine-readable way using ontologies such as PROV Ontology (PROV-O)^65^ and Provenance, Authoring, and Versioning (PAV).^66^

##### Community-endorsed metadata (FsF-R1.3-01M)

This metric (FsF-R1.3-01M) verifies if the metadata of a data object are available following community-endorsed metadata standards available through the RDA community-maintained Metadata Standards Catalog. The test compares the namespaces included in all retrieved metadata documents with the namespaces of domain metadata standards listed in the RDA catalog. If the data provision service (e.g., OAI-PMH and CSW endpoints) of the repository hosting the object is not supplied as part of the assessment request, then there should be a mechanism to infer this information based on the object identifier provided. Although registries such as FAIRsharing^67^ and re3data^21^ list metadata access endpoints of data repositories, these endpoints cannot be identified automatedly, starting from an object identifier. DataCite has started mapping the client (data provider) identifiers collected as part of the PID registration to the re3data repository identifiers. We use the mapping to automatically discover metadata access endpoints.

##### Community file formats (FsF-R1.3-02D)

The use of file formats designed for long-term archiving is critical to facilitate long-term data reusability.^68^ A variety of discipline-specific and multidisciplinary file formats have been developed during the last decades, which are widely used within and beyond distinct scientific domains. Taking these into consideration, the practical test validates the format of a data object in terms of different aspects, such as long-term file format, open file format, and scientific file format.

### Technical implementation

**Figure 3:**
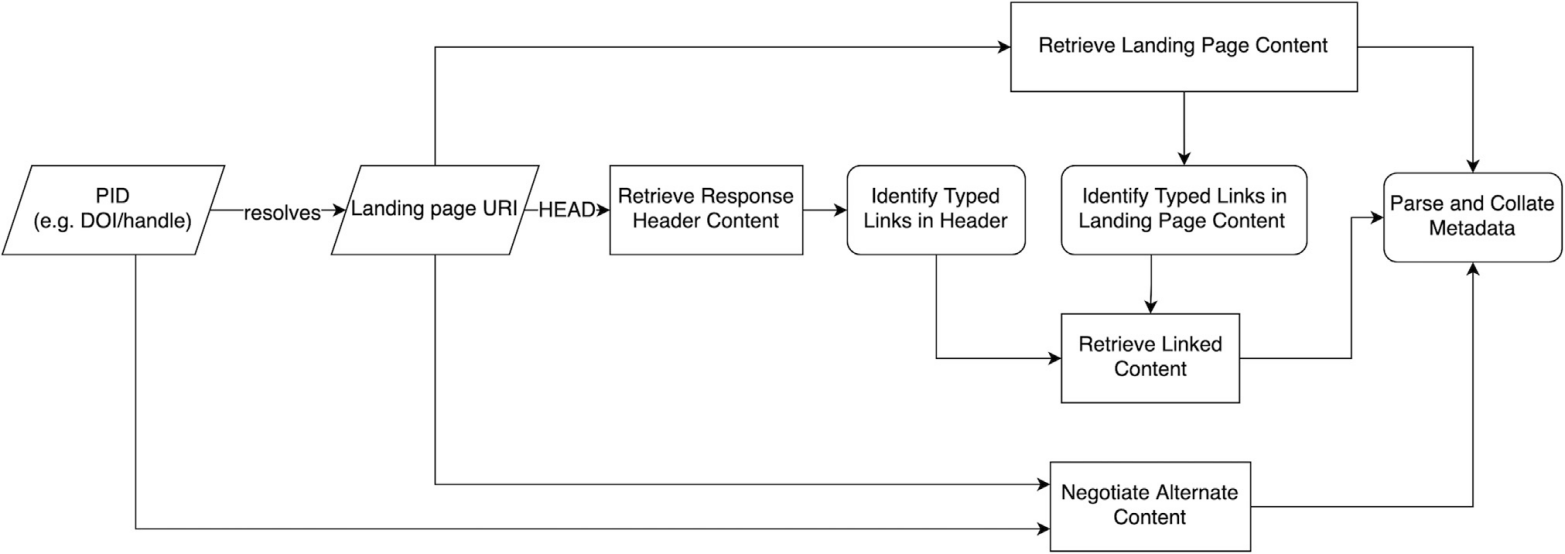
Metadata gathering via standard methods

**Figure 4:**
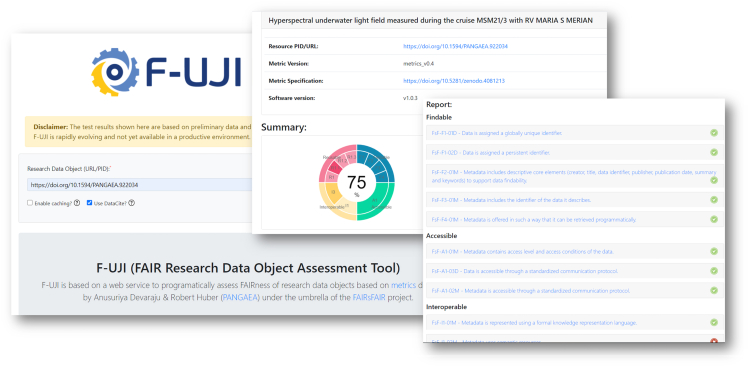
The front end of F-UJI

F-UJI is available as a REST API and is described using the OpenAPI Specification. We released it under the MIT License.^9^ The tool accepts two inputs: the unique identifier of the data object to be evaluated, and, if available, the repository's metadata provision service (e.g., OAI-PMH and CSW). These protocols are of particular interest since they are commonly used within the scientific community to exchange metadata in cross-disciplinary and community-specific formats. Figure 3 illustrates the initial stage of the assessment workflow: collecting and compiling metadata that are used for subsequent tests. The assessment comprises several primary technical operations detailed in sections “identifier-based metadata extraction” to “FAIR-enabling services.” To help users interpret the assessment results, the tool produces a JSON file containing the assessment results, including scores, practical tests, inputs and outputs, and assessment contexts for each of the metrics. Figure 4 shows the front end of F-UJI and an example of the assessment report of a data object. In addition to the online report, the tool renders a FAIR badge to provide an intuitive summary of FAIR aspects of the object evaluated.

#### Identifier-based metadata extraction

The tool first checks the identifier provided in terms of its syntax. For this purpose, it uses the idutils Python library to identify standard identifier schemes. The library includes an extensive list of regular expressions to match common identifier patterns and syntax. In addition, it recognizes identifiers that comply with identifiers.org-supported schemes.^69^ The tool further evaluates if the identifier can be transformed into an actionable representation (e.g., 10.1594/PANGAEA.810463 into https://dx.doi.org/10.1594/PANGAEA.810463), and then evaluates if the identifier resolves to a page on the Web, a prerequisite to extract metadata. F-UJI retrieves the metadata of a data object via the mechanisms below:

• The tool parses metadata embedded in the landing page following the conventions for Dublin Core encoded in HTML,^52^ the rules for RDFa Core,^70^ the microdata syntax,^71^ the OpenGraph protocol,^28^ and the Schema.org JSON-LD representation.^72^
• The tool uses the Signposting approach to access related resources, including the object's metadata document. Typed links are offered in HTTP Link headers or additionally through the HTML link elements.
• The tool retrieves the metadata representation in different formats (e.g., JSON, RDF) using content negotiation.

The tool includes dedicated parsers for processing metadata represented in domain-agnostic standards such as Dublin Core, schema.org, DataCite, and DCAT in appropriate formats (XML, RDF, or JSON) and is extensible to incorporate new parsers. We utilize the RDFLib Python package for processing RDF-metadata documents and use SPARQL queries to retrieve Dublin Core and DCAT-2 metadata. To parse XML documents, we implemented a set of domain-specific XML-based parsers for some of the most frequently used metadata standards such as Metadata Encoding & Transmission Standard (METS), MODS, DDI Codebook, and ISO 19115:2003: Geographic information – Metadata. F-UJI collates the metadata and then applies it to evaluate the data object against the metrics. Additionally, all namespaces and schema indications found within the above-mentioned linked data offerings and metadata formats are collected and stored by F-UJI to assess if semantic resources are applied (FsF-I1-02M).

#### Assessment over data content

F-UJI uses several ways to determine the object's content identifiers. For example, through an appropriate metadata field defined in schemas such as DataCite, schema.org, RDF-based metadata are provided using the DCAT vocabulary or data content identifiers offered via the signposting links. It uses Apache TIKA to analyze the downloaded data files. It verifies if the data content descriptors (e.g., data format, size, variables) specified in the metadata are an actual reflection of the actual data deposited. Most domain-agnostic metadata standards do not represent measured variables. Therefore, the tool at present uses the schema.org property (https://schema.org/variableMeasured), and this limitation is communicated as part of the object's assessment result. Further, F-UJI verifies a data object's resource type (FsF-F2-01M) through the controlled vocabularies for resource types provided by schema.org, DataCite, and DublinCore.

#### FAIR-enabling services

F-UJI utilizes several FAIR-enabling services, which we categorized into two types:

• Services are used to validate the descriptions (i.e., metadata and namespaces) of a data object, such as SPDX License List, RDA Metadata Standards Catalog, and LOV, and Linked Open Data Cloud. The tool applies fuzzy string matching to compare and determine the degree of the similarity between the original description specified (e.g., license name) and the value listed in an external service (e.g., SPDX License List).
• Services are used to gather metadata of a data object and its contexts. Consider, for example, the PID providers such as DataCite or identifiers.org support services that offer metadata of data objects in various formats such as JSON-LD and XML via content negotiation. F-UJI identifies the domain-specific metadata offerings (FsF-R1.3-01M) based on the metadata harvesting protocols implemented by the repository of interest, such as OAI-PMH (ListMetadataFormats request) or OGC CSW (GetCapabilities request). Suppose the user gives no standard metadata endpoint as part of the object's assessment request. In that case, F-UJI tries to discover existing metadata endpoints (e.g., based on SPARQL, OAI-PMH) offered by a data repository using the re3data API. In detail, F-UJI requests the re3data API with a previously identified DataCite repository identifier. The repository identifier was supplied by the repository when registering the object with a PID through DataCite.

In developing a sustainable FAIR assessment tool, it is essential to apply FAIR-enabling services that are open, well governed, and accessible in a standard way. Although related services exist, they do not fully address the FAIR assessment requirements, and this led us to develop controlled vocabularies as part of the F-UJI implementation. For example, some services (e.g., the PRONOM registry or FILExt) provide extensive lists of file formats and associated mime types. However, these registries do not indicate the target scientific community of the formats. Therefore, we collected discipline-specific formats from static Web pages such as the Archive Team list of scientific file formats^73^ or Wolfram Alpha list of supported scientific file formats^74^ and the Library of Congress Recommended Formats list.^75^ We collated existing file formats with the lists above, which resulted in a controlled list of scientific formats that includes mime types and applicable scientific communities. Further, we assembled two extensible, controlled lists dedicated to long-term and open formats. The controlled list of long-term file formats is based on the ISO/TR 22299 standard for digital file format recommendations for long-term storage.^35^ The open file format list largely relies on the Wikipedia list of open formats and the list collected by the openformats.org initiative. The practical test uses the three controlled lists to assess an object's file format based on its mime types.

### Evaluation and results

This section describes the FAIR evaluation of the data objects published by the pilot repositories. We applied the release (v1.0.0) of the tool to perform the evaluation.

#### Data objects and pilot repositories

**Figure 5:**
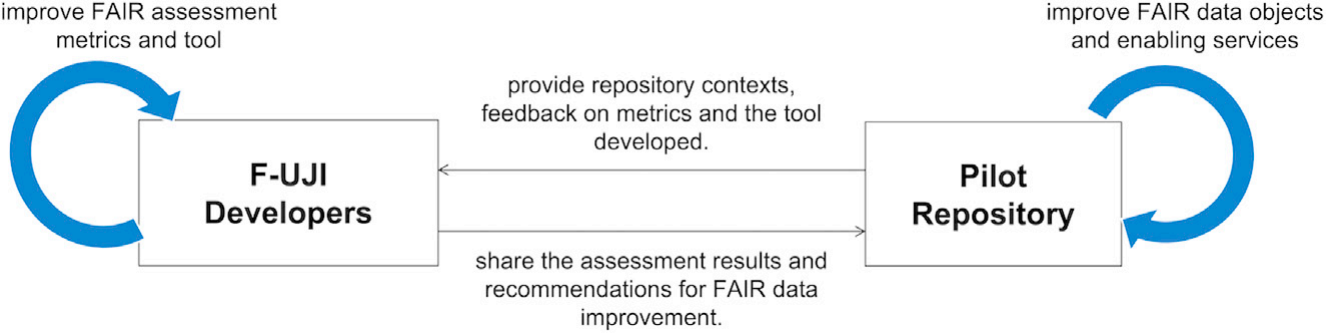
Repositories in the loop

Figure 5 summarizes the interaction between F-UJI development team and the pilot repositories (Table 2) selected for in-depth collaboration with the project. During the first iteration, we worked with the repositories to identify the scope of data objects to be tested and repository contexts (e.g., metadata services and standards). Next, we evaluated 500 random objects based on predefined criteria from each repository, and the requirements vary from one repository to another. For instance, in DataverseNO, data objects were selected based on research topics, whereas in Phaidra-Italy, the data objects were selected based on object types (image, book, collection, video). The test collections also include both new and old data objects. Finally, we developed recommendations for each repository to improve FAIR data based on the assessment conducted. We discussed the results and the recommendations with the repositories and then identified critical areas of data improvement. This prioritization is essential due to the limited development resources available at the repositories. The consultation and automated assessment process have motivated all the pilot repositories to improve their data objects and associated services. Simultaneously, based on the feedback from the repositories, we fine-tuned the tool and the metrics.

**Table 2:** Pilot repositories

Data repository	Subject areas	Repository URL
PANGAEA	earth and environmental sciences	https://pangaea.de/
CSIRO Data Access Portal	multiple disciplines	https://data.csiro.au/collections
DataverseNO	multiple disciplines	https://dataverse.no/
WDCC CERA	Earth system science	https://cera-www.dkrz.de/
Phaidra-Italy	cultural heritage	https://phaidra.cab.unipd.it/

## Results

**Figure 6:**
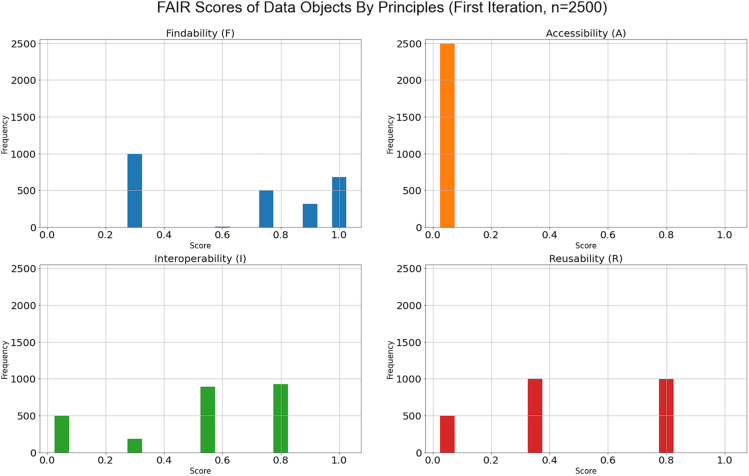
FAIR scores of data objects resulting from the first test iteration

**Figure 7:**
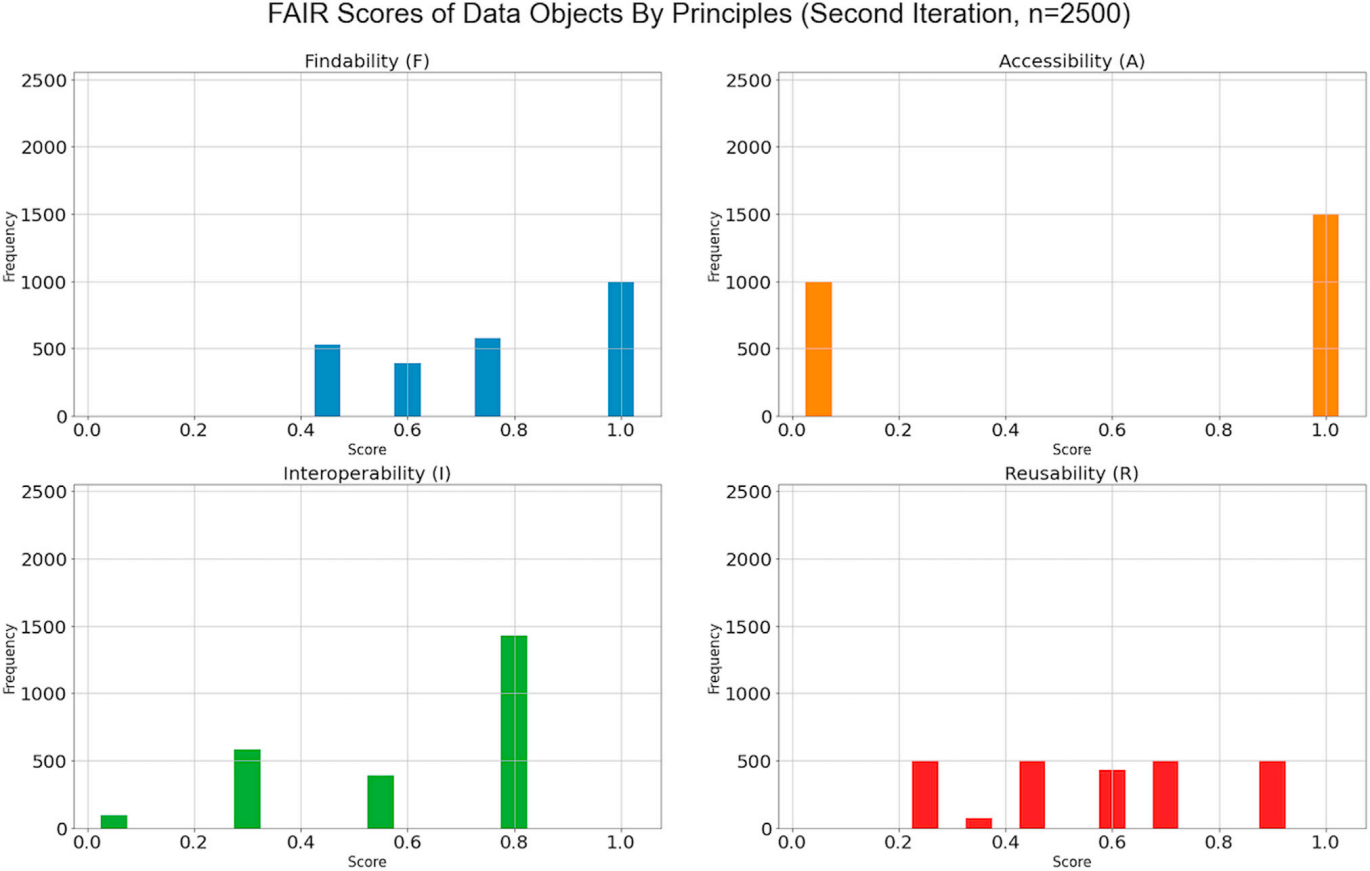
FAIR scores of data objects resulting from the second test iteration

We assessed the objects in two iterations. The assessment is based on the 13 out of 17 metrics listed in Table 1, as we have only implemented 13 metrics at the time of the first assessment. The charts (Figures 6 and 7) represent the FAIR scores of 2,500 data objects tested (500 objects from each of the repositories) by each principle, before and after FAIR data improvement. For data supporting the figures, see Devaraju and Huber.^76^ The horizontal axis represents the data objects' FAIR score, and the vertical axis indicates the number of objects tested. The FAIR score ranges from 1 (highest) to 0 (lowest). When analyzing the results, we consider the score 0.5 and above as an average level of FAIRness, and a score of 0.8 and above is exceptional.

During the first test iteration (Figure 6), more than 50% of the total objects tested scored higher than 0.5 in findability and interoperability. Sixty percent of the total objects have a score below 0.5 for the reusability principle. We observed the lowest scores for all the objects for the accessibility principle. The reason is that the principle was tested in terms of only one metric (FsF-A1-01M) that was not met by most of the repositories. This metric requires a data object's access level and conditions to be explicitly specified as part of the object's metadata.

The second iteration (Figure 7) was performed after the repositories improved the metadata of the objects and related services. It can be observed that, during the second test iteration, the objects achieved a huge score improvement at all principles, with 60% of the total objects or more objects having scores higher than the average FAIR score (0.5). Specifically, 40% of these objects are at or above the exceptional score at findability, accessibility, and interoperability principles. Three out of the five repositories have implemented the requirement for FsF-A1-01M, resulting in 60% of the total objects having full scores at the accessibility principle. Concerning the reusability principle, the scores exhibit a slight variation between the first and second iteration. The reason is that one of the repositories excluded the metadata standards supported from its metadata provision (OAI-PMH) endpoint. Consequently, the tool could not test the repository's objects against the metric FsF-R1.3-01M. This finding suggests the importance of incorporating a data repository holding the objects in the evaluation process as the repository's objects and services may develop over time. To track the evidence of change, the assessment report generated by the tool (Figure 4) captures the object's identifier, assessment date, and version information in addition to the input, output, and contexts of each of the metrics evaluated.

## Discussion

This section summarizes observations and insights gained from the development of the FAIR metrics and the automated assessment tool.

### From principles to metrics

• Measuring data objects based on the FAIR principles in practice is not straightforward for several reasons. On the one hand, evaluation methods on the aspects (e.g., rich, plurality, accurate, relevant) specified in the principles still require human mediation. On the other hand, clear and machine-assessable metrics are essential to assess data objects programmatically. Our approach when developing FAIR metrics is to improve data reuse instead of adhering to the literal sense of the principles. For example, the over-simplistic assumption of registering data and metadata objects with permanent identifiers (principle F1) is not in line with current PID practice,^37^ and this will influence the automated assessment. Criteria for a FAIR vocabulary (principle I2) require further clarification. Preserving a data object's metadata (principle A2) depends on its data repository's preservation practice; hence, it should be addressed at the repository level.
• The data assessment based on F-UJI is centered on core metrics until domain- or community-specific FAIR criteria are agreed upon (e.g., in terms of domain-specific schemas and practices to support FAIR data). For this reason, we designed the practical tests in consideration of generic domain-agnostics metadata standards. Ideally, the core metrics should be extended with metrics that reflect discipline requirements.
• The consultation with the pilot repositories suggests the importance of expanding current metrics with the quality and usability aspects of research data, which were not explicitly addressed by the FAIR principles. These aspects play an essential role in data reuse and management.^77^^,^^78^

### FAIR-enabling services and repositories

• In the FAIR ecosystem, FAIR assessment must go beyond the object itself.^79^ FAIR-enabling repositories should evolve in parallel. The FAIR metrics can be applied to assess if a data repository strives toward FAIR data objects. The trustworthy repository requirements, e.g., developed by CoreTrustSeal,^80^ are essential to ensure the objects are preserved and remain FAIR over time.
• Our approach combines an automated FAIR object assessment with an iterative consultation process involving repositories hosting the objects to incorporate their feedback. This approach's outcomes suggest the importance of repositories in enabling FAIR assessment and the improvement of data based on the assessment, and this should be recognized and appreciated.
• FAIR-enabling services play a vital role in an automated FAIR data assessment. Some services provide further inputs to run the practical tests. In contrast, others are “lookup” services needed to validate the metadata and contexts of the object. We believe FAIR data assessment is a continuous improvement process. Thus, it is crucial to adopt standardized, open, machine-friendly, and well-governed services to support the long-term data assessment. The section “fair data object assessment metrics” has highlighted the lack of machine-readable, authoritative lists of persistent identifier systems; community-specific metadata standards; and communication protocols for which promising approaches are now emerging, such as the FAIRsharing initiative.^67^

### Assessment depth and extent

Several factors influence the depth and extent of the automated assessment:

• A data object may be registered with a persistent or non-persistent identifier. With a non-persistent identifier like URL, the automated assessment is limited to metadata embedded on the data page. In contrast, through a PID, the information needed to assess the object (e.g., metadata of the object and its repository contexts) can be retrieved automatedly in various ways; e.g., through content negotiation, structured data in the data page, and the object's PID provider.
• A data object may be complex; e.g., a collection may consist of one or more data series. These entities may have different coverage of metadata, and their types and relation may not be explicitly specified as part of the metadata. This may affect the assessment results, especially when the objects to be evaluated are selected randomly. For instance, in the World Data Center for Climate (WDCC) Climate and Environmental Retrieval and Archive (CERA) repository, DOIs are provided at both experiment (i.e., collection) and dataset levels. Some metadata are provided at the experiment (collection) level (e.g., contact person, model description quality documentation), and other metadata are provided at the dataset level (e.g., access and usage constraints). Future work should consider the object types as well as their relations so that the tool can navigate from a parent object to its child object (vice versa), and then run the assessment based on the types of the objects.
• Research data should be as open as possible and as closed as necessary.^3^ In its current form, the tool evaluates if the metadata include the access level and the conditions by which the data are accessible and relevant communication protocols. There may be procedures to manage and limit access to restructured data for legitimate reasons,^81^ and they may vary by repositories. In the current implementation, the automated tests only inspect restricted data against specific metrics that depend wholly on a data object's metadata, not the data content files (e.g., FsF-A1-03D, FsF-R1-01MD). Further research is needed in the context of negotiating secure access and performing an automated FAIR assessment over restricted data.
• One of the technical challenges in automating a data object's assessment is analyzing many data files. For example, the data object https://doi.org/10.18710/WYV6PR comprises 1,093 individual files. It is not feasible to analyze all the object files; e.g., to assess the object against the metrics FsF-A1-03D, FsF-R1.3-02D, and FsF-R1-01MD. For pragmatic reasons, the tool selects a subset of the files randomly. The subset's size is configurable through the tool, and the information of the analyzed files is shared as part of the assessment report.

### Transparency

One crucial aspect of the FAIR data assessment, which is overlooked by existing assessment frameworks, is communicating the assessment results to users transparently. The assessment report generated by the tool offers a detailed provenance of the practical tests applied on a data object (e.g., what, and how the object is measured) and is accompanied by sufficient contextual information of the assessment. The pilot repositories highly appreciated this aspect of the tool as it helps them identify key results and critical areas of the FAIR data improvement.

### Conclusions

Investigating FAIR metrics and applying them in practice have been continuing concerns in research data publication. We believe our work contributes to this strand building a set of core metrics to assess the FAIRness of scientific data objects. At the same time, it offers an open-source tool (F-UJI) to apply the metrics in practice. We elaborate the core metrics in terms of practical tests that follow existing research data standards and sharing practices. The metrics are built on established work and improved gradually through iterative feedback from various stakeholders. The assessment tool went through iterations of testing with actual data objects and was enhanced based on feedback gathered from the pilot repositories. The pilot assessments' findings indicate the importance of considering the contexts (repositories' procedures and practices) as part of the assessment.

To enable in-depth FAIR data assessment, we interface F-UJI with several open FAIR-enabling services, and this integration has not yet been explored thoroughly by existing FAIR frameworks. Resources retrieved from the services are cached as part of the tool to speed up the assessment. The source code of the tool is accessible from a public repository and available for reuse. F-UJI is built with Open API to reduce the barriers for machines to understand the service's capabilities. For ease of use, we set up a front end on top of the API, enabling users to test their datasets on the Web. Using the F-UJI front end, users will be able to explore different facets of an assessment report. For ease of use, the report includes a FAIR badge summarizing the FAIRness of a data object assessed. The report consists of descriptions of the practical tests, including the assessment contexts. In other words, the conditions under which the objects were evaluated are made transparent to users. These details will help the users interpret the assessment results and help them identify areas where improvements required.

The metrics and the assessment tool have received positive feedback from several projects and repositories in EOSC. The ongoing pilots that we are aware of include the assessment of data objects in the context of EOSC projects (e.g., EOSC Synergy, EOSC Nordic, NI4OS-Europe, ARCHIVER), consortia (e.g., ICOS, EMSOdev, CESSDA, NFDI4Chemistry) and repositories (e.g., DataverseNL). We plan further pilots with the repositories who have expressed their initial interests in testing the tool, including OpenAIRE Advance, Copenhagen University Library, and Archaeology Data Service.

## Experimental procedures

### Resource availability

#### Lead contact

Further information and requests for resources on the assessment should be directed to and will be fulfilled by the lead contact, Anusuriya Devaraju (a.devaraju@uq.edu.au).

#### Materials availability

This study did not generate new unique reagents.

## Data Availability

The source code of the tool developed (F-UJI) is available through https://github.com/pangaea-data-publisher/fuji. The assessment results have been deposited at Zenodo under https://doi.org/10.5281/zenodo.5302018 and are publicly available as of the date of publication.
